# Crafting medical MCQs with generative AI: A how-to guide on leveraging ChatGPT

**DOI:** 10.3205/zma001675

**Published:** 2024-04-15

**Authors:** Matthias Stadler, Anna Horrer, Martin R. Fischer

**Affiliations:** 1 LMU University Hospital, LMU Munich, Institute for Medical Education, Munich, Germany

**Keywords:** medical education, generative AI, ChatGPT, MCQ creation, prompt engineering

## Abstract

As medical educators grapple with the consistent demand for high-quality assessments, the integration of artificial intelligence presents a novel solution. This how-to article delves into the mechanics of employing ChatGPT for generating Multiple Choice Questions (MCQs) within the medical curriculum. Focusing on the intricacies of prompt engineering, we elucidate the steps and considerations imperative for achieving targeted, high-fidelity results. The article presents varying outcomes based on different prompt structures, highlighting the AI's adaptability in producing questions of distinct complexities. While emphasizing the transformative potential of ChatGPT, we also spotlight challenges, including the AI’s occasional “hallucination”, underscoring the importance of rigorous review. This guide aims to furnish educators with the know-how to integrate AI into their assessment creation process, heralding a new era in medical education tools.

## Introduction

Medical education, in its pursuit of equipping future practitioners with essential knowledge and skills, often places heavy responsibilities on academic staff. This dedicated group of professionals juggles a myriad of tasks, from direct patient care and research to administrative duties and, notably, the preparation and oversight of student examinations [1]. A constant expectation looms over these educators to ensure that assessments are of high quality, fair, and properly aligned with set learning objectives [2], [3]. This demanding standard, combined with the challenges of contemporary medical education, accentuates the need for innovative solutions.

Crafting high-quality multiple-choice questions (MCQs) for assessments, a traditional and widely-accepted format, has been known to be both time-consuming and labor-intensive. Despite the recognized benefits of MCQs – such as their objectivity, cost-effectiveness, and comprehensive content coverage [4] – creating them demands a keen eye and considerable expertise. This intricacy often amplifies the already substantial workload for medical educators.

The burgeoning field of artificial intelligence (AI), specifically within the realms of machine learning and large language models, hints at promising solutions to this enduring challenge [5]. The ChatGPT model, developed by OpenAI [6], emerges as a notable contender with its proven capabilities in generating coherent, diverse, and human-like text. The potential of leveraging such a tool to draft MCQs, thereby alleviating the challenges faced by medical educators, remains an intriguing and possibly transformative prospect for the world of assessment in medical education. Notwithstanding this potential for simplified question creation, the steps for assuring the quality of high-quality written examinations at medical schools remain fully valid and should apply [7]. 

In this short How-to article, we aim to demonstrate the potential of crafting medical exam questions using large language models. In that, we provide an illustrative example, discussing the necessity of carefully engineering the necessary prompt to the AI. 

## Utilizing generative AI in crafting medical MCQs

Generative AI has been steadily permeating the landscape of medical education, with models like ChatGPT taking the lead in showcasing the potential of AI in this domain. Recent studies emphasize ChatGPT’s impressive capabilities, both in processing intricate medical information and in generating complex clinical content [8], [9], [10]. Particularly notable is its marked improvement in performance metrics, as observed in biomedical and clinical questions. In comparisons with previous versions, the latest iteration of ChatGPT demonstrates a surging accuracy, even outstripping established benchmarks like the United States Medical Licensing Examination (USMLE), a standardized series of exams that assess the medical knowledge and clinical skills of individuals seeking to practice medicine in the United States, by crossing the 60% threshold in specific instances [11].

Furthermore, the model’s general content foundation, as opposed to domain-specific counterparts like PubMedGPT, gifts it a broader spectrum of clinical information to pull from, leading to more definitive and congruent responses [11]. 

### A potential solution for MCQ generation 

Given ChatGPT's demonstrated capabilities in medical knowledge comprehension and generation, its application in crafting MCQs for medical exams seems promising [5]. Its vast knowledge base, combined with the insights from its test performance, can be harnessed to generate questions that are not only relevant but also aligned with the complexities of contemporary medical education. Furthermore, by merging foundational models like ChatGPT with domain-specific LLMs or other medical resources, we might unlock a synergistic tool that efficiently crafts high-quality MCQs, alleviating the traditional burdens faced by medical educators [10].

The efficacy of the generated response is profoundly influenced by the precision and clarity of the input prompt [12], [13]. Prompt engineering is a critical aspect of harnessing the power of generative AI systems. It involves crafting well-defined and contextually relevant input queries or instructions to achieve desired output and needs initial refinement of input prompts in order to get the intended output [14]. Effective prompt engineering requires a deep understanding of the model's capabilities and limitations, as it serves as the bridge between human intention and machine response. In the realm of generative AI, prompts can range from simple queries to more complex, multi-step instructions. They can be used for a wide array of applications, from content generation and translation to problem-solving up to creative storytelling. 

In the domain of assessment in medical education, this precision is paramount, given the complexity and nuanced nature of the subject matter. A well-crafted prompt can lead the model to produce accurate, relevant, and high-quality medical exam questions. However, there are instances where even with a detailed prompt, the AI might “hallucinate”, generating details or facts not rooted in its training data, adding an unforeseen layer of complexity. In contrast, a vague or imprecise prompt may not only result in outputs that are tangential but also amplify the risk of these hallucinations, producing content lacking the necessary specificity for a rigorous medical assessment. Thus, while prompt engineering offers potent guidance, it is imperative to rigorously review AI-generated content for both accuracy and relevance in the context of medical education. Several factors contribute to the challenges faced in prompt engineering:

1. **Complexity of medical terminology: **Medical education is characterized by its intricate terminology and the interrelatedness of concepts. Crafting prompts that encapsulate this complexity while ensuring they guide the AI in the right direction requires a deep understanding of both the subject matter and the model’s functionalities.
2. **Dynamic nature of medical knowledge:** As medical science continuously evolves, prompts must be updated and refined to reflect the most current knowledge and practices. This dynamic nature necessitates constant vigilance and recalibration of prompts.
3. **Contextual ambiguity:** Medical scenarios often involve multi-faceted problems with overlapping symptoms or treatments. Ensuring that prompts lead to distinct, clear, and unambiguous responses is a meticulous endeavour.
4. **Balancing brevity with clarity:** While it’s crucial to provide the AI with adequate context, there’s a risk of overloading the prompt with excessive information. Striking a balance between brevity and clarity is an art that needs to be mastered for effective prompt engineering. Too vague a prompt may result in unpredictable or irrelevant responses, while being overly prescriptive can stifle creativity and limit the AI’s ability to adapt to nuanced tasks.

In the following part of this article, we will illustrate how these factors need to be considered in order to engineer a prompt to gain the desired outcome. Additionally, we will provide a blueprint for creating MC-items to assess diagnostic knowledge in a clinical internal medicine exam.

### Prompt engineering illustrated: crafting an effective MCQ generation prompt

**Figure 1:**
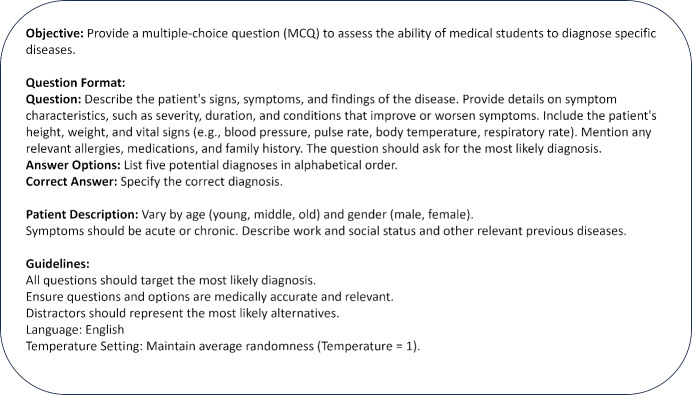
Prompt to generate multiple-choice question (MCQ) to assess the ability of medical students to diagnose specific diseases

In the evolving landscape of medical education, the precision of AI-generated assessments depends significantly on the quality and clarity of the input prompt [12]. To illustrate this concept, we deconstruct a sample prompt designed for generating Multiple Choice Questions (MCQs) that assess the diagnostic abilities of medical students. Figure 1 (Fig. 1) provides a prompt engineered to generate a multiple-choice question (MCQ) to assess the ability of medical students to diagnose specific diseases.

The provided prompt is distinctly structured, deviating from the typical conversational interactions most users have with generative AI. While it may not embody the naturalness of everyday communication, such a structured format facilitates clearer, more precise instructions to the AI. This not only aids in achieving the desired output but also simplifies the process of identifying and adapting specific sections of the prompt for further refinement. Delving deeper, the salient components of the prompt include:

1. **Objective:** This directive establishes the primary intent behind the task. By highlighting the goal of crafting an MCQ tailored for medical diagnostic skills, it ensures the focus remains on creating questions that are suitable for medical evaluations.
2. **Patient description:** This sets the backdrop for the questions. Offering variations in the patient's demographics, previous diseases, and social and work status introduces diversity in potential medical scenarios. This component ensures that each MCQ can be tailored to address a myriad of medical presentations and conditions, considering age, gender, and history.
3. **Question format:** Serving as the blueprint for the AI, this section lays out the expected structure for every MCQ.
  – *Question:* This section ensures a comprehensive presentation of the patient, capturing all essential details from symptoms to vital stats.
  – *Answer options:* By asking for a list of potential diagnoses, it ensures a structured platform to assess differential diagnostic capabilities.
  – *Correct answer:* Provides the AI with a directive to pin down a definitive diagnosis, offering clarity and precision to the MCQ.
4. **Guidelines:** This section serves as the quality control mechanism. It provides overarching directives to ensure that the MCQs remain relevant, medically accurate, and challenging.
  – *Diagnostic focus:* By underlining the significance of the most probable diagnosis, the AI’s output is ensured to mirror real-life clinical situations.
  – *Accuracy and relevance:* A critical safety net, ensuring that the generated MCQs remain aligned with current medical knowledge and best practices.
  – *Distractors:* Emphasizes the need for plausible, yet challenging alternative options in the MCQs, thereby testing the nuanced understanding of the responder.
5. **Language:** A straightforward directive, it specifies the linguistic output. While set to English here, it allows for adaptability to different language requirements.
6. **Temperature:** A pivotal parameter when working with generative AI models. At a setting of temperature=1, it ensures the generated MCQs strike the right balance – they are diverse, inventive, yet medically precise and consistent.

Attachment 1 provides examples of how changes in this prompt would change the provided output when given to GPT-4. The original prompt results in a traditional MCQ, offering a meticulous patient history followed by a diagnostic query. This structure and content arguably occupy a mid-tier difficulty level, aligning with general medical education standards. The rare disease-focused prompt, while retaining a semblance of the original’s structure, navigates into more specialized terrains of medicine. Its depth and specificity elevate its complexity, posing a greater challenge to students by requiring nuanced clinical acumen. Conversely, the pediatric patient-focused prompt, though tailored to a younger demographic with age-specific symptoms, maintains a foundational complexity. While it necessitates knowledge of pediatric conditions, it doesn’t veer deeply into sub-specialties, making it approachable for a broader audience. The succinct output, stemming from a prompt devoid of granular details, is reminiscent of swift clinical decision-making scenarios. Its brevity might be construed as simplifying, but it simultaneously demands a sharp clinical eye, rendering its difficulty equivocal. Lastly, the prompt inducing randomness is inherently unpredictable in difficulty. Its divergence from structured guidance can spawn questions ranging from elementary to esoteric, underscoring the capricious nature of unguided AI outputs. 

In addition to generating individual questions, we have combined the original prompt (see above) with a prompt for generating a written exam consisting of two structured questions for each of ten given differential diagnoses symptom of respiratory distress. The result of the automatically generated written exam can be found in the electronic supplement [https://osf.io/2bg9p/]. It shows that full exams can be created by the carefully prompted AI with little effort using simple blueprints. Using generative AI systems for creating exam questions can increase efficiency but requires careful planning and oversight to ensure that the AI-generated questions meet educational requirements context specific factors, e.g., differences in the output regarding the differences in temperature in the AI-generated output.

## Discussion

The potential of AI for assessment in medical education, specifically in the generation of MCQs, reflects the progress of machine learning and computational advancements. ChatGPT, with its vast knowledge base and adaptability, provides a significant solution, especially as medical educators face increasing demands [3], [9]. 

However, the quality of AI-generated MCQs relies heavily on effective prompt engineering [12]. Producing prompts that guide ChatGPT to generate relevant and contextually correct outputs necessitates a deep comprehension of both the medical domain and the AI model’s mechanics. While AI offers numerous advantages in this field, it is crucial to use its capabilities alongside a comprehensive understanding of the medical subject matter. The challenge lies in balancing the intricacies of medical knowledge with AI and human curation to assure quality. Given the continuous advancements in medical science, the inherent complexities of medical terminology, and the need for concise yet clear prompts, crafting the perfect prompt is a daunting task. Our example offers a practical guide, shedding light on the challenges faced while framing an effective MCQ generation prompt. We illustrate, how even minor changes in the prompt will influence not just the intended content but can completely reframe the output.

An essential point of consideration is the notion of ChatGPT’s “understanding”. While the model can provide accurate outputs, it doesn’t “understand” in the way humans do. It generates responses based on patterns identified during training. Given the critical nature of medical education, this distinction is vital. Additionally, the vast training data of ChatGPT, while enabling a broad knowledge spectrum, might introduce potential biases. Any bias present in the training data could be reflected in its outputs, warranting careful scrutiny and periodic assessments with critical human curation by medical subject-matter and assessment experts. With the integration of AI into education, many questions about responsibility and accountability arise. If an AI-generated MCQ or total exam is flawed or unintentionally misleads, the responsibility becomes a gray area [15].

## Conclusion

The integration of AI into medical education, as demonstrated in our examination of MCQ generation using ChatGPT, is both promising and complex. While AI offers the potential to assist and alleviate some burdens from medical educators, it’s not a standalone solution. The key to harnessing AI’s capabilities lies in meticulous prompt engineering and a thorough review by individuals with deep medical content knowledge. As we navigate the future of medical education, merging AI capabilities with human expertise will be vital. With continued research, ongoing refinement, and collaboration between disciplines, the future holds a synergy where educators are better equipped, and students benefit from a more comprehensive and detailed learning experience.

## Note

During the preparation of this work the authors used GPT4.0 to generate multiple choice questions, as described in the paper’s methods section. Further, GPT4.0 was used in writing and language editing of the paper. After using this tool, the authors reviewed and edited the content as needed and take full responsibility for the content of the publication.

## Authors’ ORCIDs

- Matthias Stadler: 0000-0001-8241-8723
- Anna Horrer: 0000-0003-1029-5896
- Martin R. Fischer: 0000-0002-5299-5025

## Competing interests

The authors declare that they have no competing interests. 

## Supplementary Material

Examples
