# Supplementary material for: Crafting medical MCQs with generative AI: A how-to guide on leveraging ChatGPT
Source: GMS J Med Educ. 2024 Apr 15;41(2):Doc20. doi: 10.3205/zma001675 (PMC11106576; doi:10.3205/zma001675)
Supplement: Examples [file JME-41-20-s-001.pdf]

Attachment 1: Examples

Changes from the original prompt	Output
Original prompt	**Patient Description**: A 25-year-old female presents to the clinic. She reports feeling fatigued, experiencing unexplained weight gain, and feeling cold constantly over the past 6 months. On further questioning, she also mentions a decrease in appetite and constipation. She works as a bank clerk and denies any substance abuse or significant stressors in her life. Her mother had similar symptoms at a young age. Her vital signs are: Blood pressure 120/80 mmHg, pulse rate 55 bpm, body temperature 36.2°C, and respiratory rate 12/min. She has no known drug allergies.<br>**Question**: Based on the clinical presentation and history, what is the most likely diagnosis for this patient?<br>**Answer Options**: A) Addison's disease B) Anemia C) Hashimoto's thyroiditis D) Hypoparathyroidism E) Polycystic ovary syndrome (PCOS)<br>**Correct Answer**: C) Hashimoto's thyroiditis
Specifically asking for a rare disease:<br>"Objective: Provide a multiple-choice question (MCQ) to assess the ability of medical students to diagnose **rare** diseases."	A 35-year-old male software developer presents to the clinic complaining of recurrent, painful lumps under his armpits and groin for the past 8 months. He also mentions that he recently noticed a small amount of blood on his undergarment, originating from one of these lumps. The patient has a BMI of 28, smokes 10 cigarettes a day, and does not exercise regularly. His blood pressure is 130/80 mmHg, pulse rate is 78 beats per minute, body temperature is 98.4°F, and respiratory rate is 16 breaths per minute. His past medical history is unremarkable, and he is not on any medications. There is no family history of any skin or autoimmune disorders.<br>What is the most likely diagnosis?<br>A. Furunculosis B. Hidradenitis suppurativa C. Lymphogranuloma venereum D. Lymphoma E. Tuberculosis of the skin<br>**Correct Answer:** B. Hidradenitis suppurativa
Specifically asking for paediatric patients:<br>"Patient Description: **Paediatric** patients that vary by gender (male, female). Symptoms should be acute or chronic. Describe work and social status and other relevant previous diseases."	A 7-year-old female patient is brought to the pediatrician with a 2-month history of increasing thirst, frequent urination, and unexplained weight loss. She's been less active in sports lately, saying she feels tired. The teacher also reported that she often asks to go to the bathroom during class. Her BMI is on the lower side of the normal range. No history of fever, vomiting, or abdominal pain. She is not on any medications and has no known allergies. Family history reveals that her grandmother had diabetes. The patient's vitals are:<br>• Height: 124 cm<br>• Weight: 20 kg<br>• Blood pressure: 100/62 mmHg<br>• Pulse rate: 90 bpm<br>• Body temperature: 36.9°C (98.4°F)<br>• Respiratory rate: 18 breaths per minute<br>What is the most likely diagnosis?<br>**Answer Options:** A. Diabetes insipidus B. Diabetes mellitus type 1 C. Hyperthyroidism D. Urinary tract infection (UTI) E. Cystic fibrosis<br>**Correct Answer:** B. Diabetes mellitus type 1

Attachment 1 to Stadler M, Horrer A, Fischer MR. *Crafting medical MCQs with generative AI: A how-to guide on leveraging ChatGPT.* GMS J Med Educ. 2024;41(2):Doc20. DOI: 10.3205/zma001675

Not asking for details in the question:<br>“Question: Describe the patient's signs, symptoms, and findings of the disease. The question should ask for the most likely diagnosis.”	A 25-year-old female presents with a 6-week history of fatigue, joint pain, and a malar rash. She recently began working as a teacher and has been otherwise healthy. She also mentions intermittent hair loss and ulcers in her mouth. What is the most likely diagnosis?<br>**Answer Options:**<br>A) Fibromyalgia<br>B) Lichen planus<br>C) Rheumatoid arthritis<br>D) Rosacea<br>E) Systemic lupus erythematosus (SLE)<br>**Correct Answer:**<br>E) Systemic lupus erythematosus (SLE)
Drastically increasing the output’s randomness:<br>“Temperature Setting: Maintain **very high** randomness (Temperature = **10**).”	**Age & Gender:** A 65-year-old male<br>**Work & Social Status:** Recently retired engineer, socially active in his local community club.<br>**Presenting Symptoms & Findings:** The patient reports a 3-month history of progressively worsening tremor in his right hand, which improves with rest. He also mentions feeling slightly stiff in his right arm. His daughter noticed that his face appears less expressive and he does not swing his right arm as much when he walks. No headache, weakness, or sensory changes. No history of trauma.<br>**Vitals:**<br>• Height: 5'9"<br>• Weight: 160 lbs<br>• Blood pressure: 135/85 mm Hg<br>• Pulse rate: 78 beats/min<br>• Body temperature: 98.6°F<br>• Respiratory rate: 15 breaths/min<br>**Allergies:** Penicillin.<br>**Medications:** Daily multivitamins and low-dose aspirin.<br>**Family History:** Mother had a similar tremor in her late 60s.<br>**Most likely diagnosis?** A) Essential Tremor B) Multiple Sclerosis C) Parkinson's Disease D) Peripheral Neuropathy E) Stroke<br>**Correct Answer:** C) Parkinson's Disease
